# Alterations in brain glycogen levels influence life-history traits and reduce the lifespan in female *Drosophila melanogaster*

**DOI:** 10.1242/bio.059055

**Published:** 2021-12-14

**Authors:** Deepashree Sheshadri, Akanksha Onkar, Subramaniam Ganesh

**Affiliations:** Department of Biological Sciences and Bioengineering, Indian Institute of Technology, Kanpur, Uttar Pradesh 208016, India

**Keywords:** Sexual dimorphism, Aging, Starvation resistance, Oxidative stress

## Abstract

Sexual dimorphism in lifespan, wherein females outlive males, is evident across all animal taxa. The longevity difference between sexes is controlled by multiple physiological processes with complex relationships to one another. In recent years, glycogen, the storage form of glucose, has been shown to cause rapid aging upon forced synthesis in healthy neurons. Glycogen in the form of *corpora amylacea* in the aging brain is also widely reported. While these studies did suggest a novel role for glycogen in aging, most of them have focused on pooled samples, and have not looked at sex-specific effects, if any. Given the widespread occurrence of sex-biased expression of genes and the underlying physiology, it is important to look at the sex-specific effects of metabolic processes. In the present study, using transgenic fly lines for the human glycogen synthase, we investigated the sex-specific effects of glycogen on stress resistance, fitness, and survival. We demonstrate that *Drosophila melanogaster* females with altered levels of glycogen in the brain display a shortened lifespan, increased resistance to starvation, and higher oxidative stress than male flies. The present study thus provides a novel insight into the sex-specific effect of glycogen in survival and aging and how differences in metabolic processes could contribute to sex-specific traits.

## Abstract (precis)

**Summary:** This study demonstrates that *Drosophila melanogaster* females with altered levels of brain glycogen display a shortened lifespan, increased resistance to starvation, and higher levels of oxidative stress than male flies. Thus, metabolic processes like the glycogen synthesis pathway could influence sex-specific traits in animals.

## INTRODUCTION

One of the long-standing questions in biology is the factors determining the lifespan of a species in a population (Medawar, 1952). Variations in longevity between different species and between members of the same species remain inexplicable (Weisman, 1891). In this respect, sexual dimorphism in lifespan, where females outlive males across species, including humans, is an established concept now, yet the underlying mechanisms are not fully understood (Austad, 2006, 2011; Austad and Bartke, 2015). Several theories have been proposed to elucidate the variations in lifespan between the two sexes. One is the ‘unguarded X hypothesis’, where the heterogametic sex (XY males/ZW females) is expected to have a shorter lifespan as they express deleterious alleles located on the X chromosome. The homogametic sex (XX females), in contrast, might live longer since the effect of the deleterious alleles gets diluted because of the two copies of the X chromosome (Tower and Arbeitman, 2009; Maklakov and Lummaa, 2013). Another hypothesis that explains the gender-specific difference in the lifespan is ‘maternal inheritance’, wherein maternally derived mitochondria are thought to function sub-optimally in males, thus adding to the survival advantage in females (Tower and Arbeitman, 2009; Maklakov and Lummaa, 2013). A difference in the hormone signaling pathways and metabolic circuits has also been suggested to account for the difference in the lifespan of the two sexes (Tower, 2017). Few examples include the reduction in the female lifespan post-mating by the male-specific hormone ‘sex peptide’ (Tower et al., 2017) and the shortening of male survival upon activation of the neuropeptide signaling by a female-specific pheromone (Gendron et al., 2014). Sex differences in lifespan are also linked with stress-related traits and biochemical factors (Niveditha et al., 2017). According to the ‘free radical theory of aging’ (Harman, 1992), reactive oxygen species (ROS) are one of the critical determinants of lifespan trade-offs, and a negative correlation exists between lifespan and ROS abundance in particular sex (Sohal and Orr, 2012). Studies have indicated that effective mitochondrial function and higher levels of antioxidant enzymes in females compensate for increased ROS levels (Austad and Bartke, 2015), thus earning a survival benefit over males. Other factors contributing to lifespan variations between males and females are social interaction, sex-specific genetic architecture, aging mechanisms, and fitness parameters (Tower and Arbeitman, 2009; Tower et al., 2020).

At the molecular level, studies on sex-specific lifespan have mostly looked at the difference in the expression levels of essential genes, genes coding for the hormonal and immune response, and genes involved in healthy aging (Tower et al., 2020; Tian et al., 2017). For example, a genetic perturbation in the insulin-signaling pathway increases the survival rate of *Drosophila melanogaster* females as compared to males (Clancy et al., 2001). Similarly, chronic treatment of *Drosophila* with lithium, which acts on the mechanistic target of rapamycin (mTOR) and glycogen synthase kinase-3β (GSK3β), abolishes the female advantage in lifespan with no significant changes in male survival (Zhu et al., 2015). Dietary restriction, which dramatically enhances lifespan across species, also has a pronounced effect on the female lifespan compared to the males (Magwere et al., 2004). In this context, glycogen has recently been identified as a potential regulator for the aging process (Duran et al., 2012; Sinadinos et al., 2014). Glycogen, the branched polymer of glucose, acts as an energy reservoir in animals wherein the abundance of glucose in the body activates glycogen synthase (GS), the key enzyme for glycogen synthesis (Roach et al., 2012). Interestingly, glycogen in the brain is mostly stored in astrocytes (Pellerin and Magistretti, 1994), and the glycogen content in the neurons is negligible (Saez et al., 2014). This suggests a neuron-specific adaptive response, as abnormal glycogen has been associated with Lafora disease (Parihar et al., 2018) and aberrant levels diminish the overall lifespan in the GS-overexpressing fly lines and mice models (Duran et al., 2012). In addition, neuronal GS knockdown promoted healthy aging in *Drosophila* (Sinadinos et al., 2014). Moreover, glycogen-mediated accelerated aging is seen in *Caenorhabditis elegans*, where a high sugar diet as a precursor for glycogen results in a shortened lifespan of the worms (Seo et al., 2018). The metabolic shift of glycogen synthesis from the astrocytes to the neurons in the aging hippocampus (Drulis-Fajdasz et al., 2018) and the presence of glycogen deposits in the form of *corpora amylacea* in the aging brain (Duran et al., 2019), further establish glycogen as a modifier of the aging process. We, therefore, evaluated the role of brain-specific glycogen changes as a possible factor that can influence the sex-specific lifespan in *Drosophila*. We demonstrate that *Drosophila* females with an imbalance in the level of brain glycogen display a shortened lifespan, increased resistance to starvation, elevated glycogen reserves, and higher oxidative stress than male flies. A shortening in the lifespan of females in the current study is attributed to the altered brain glycogen levels that correspond to the increased oxidative stress in the female flies.

## RESULTS

### Sex-specific effect of brain glycogen levels on lifespan in *Drosophila*

**Fig. 1.**
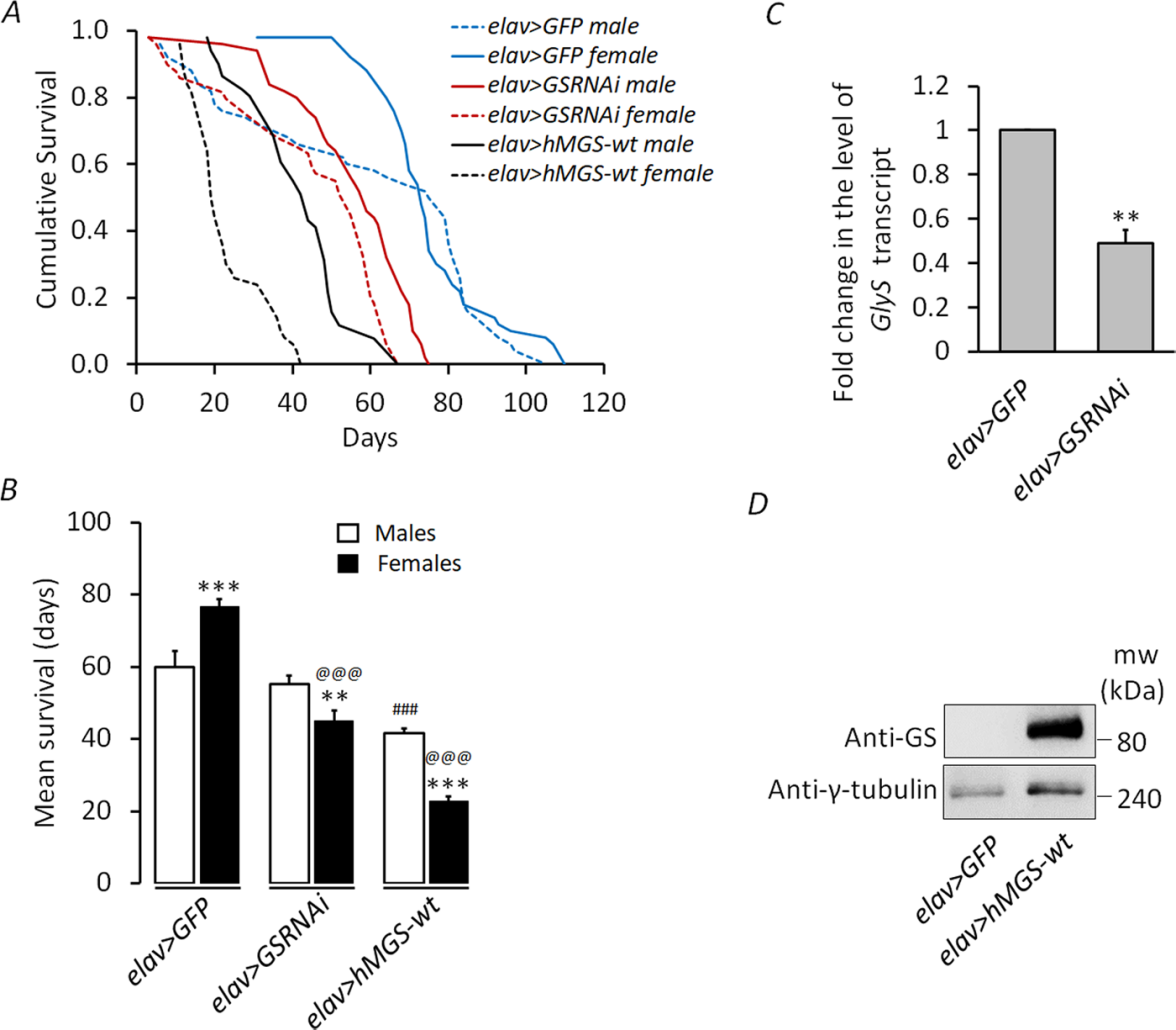
**Differential effect of brain glycogen on survival of *Drosophila* females.** (A) Kaplan–Meier survivorship graphs showing cumulative survival against time (in days) for male and female flies with pan-neuronal repression of GS (*elav>GSRNAi*) or pan-neuronal overexpression of GS (*elav>hMGS-wt*). The *elav>GFP* line served as the control. Data were analyzed using the Log-rank test using Mantel–Cox (*χ*2) and the *P*-values are shown in Table 1. (B) Bar graph representing the average lifespan (days) of flies across all sexes and genotypes as indicated (*N*=100). (C) Fold change in the transcript level of GS (*GlyS*) in the fly heads of the *elav>GFP* (control) and GS knockdown line (*elav>GSRNAi*, *N*=3). (D) Representative immunoblot showing the level of total GS in the fly heads of *elav>GFP* and *elav>hMGS-wt* line. Probing for *γ*-tubulin served as the loading control. Each bar represents the mean value±s.e. ^*^, ^#^, ^@^*P*<0.05; ^**^, ^##^, ^@@^*P*<0.01; ^***^, ^###^, ^@@@^*P*<0.001; * denotes the significance when the comparison was made between males and females of the same genotype; # denotes significance between control males and males of other experimental groups; *@* denotes significance between control females and females of other experimental groups; GS, glycogen synthase.

*Drosophila*, in laboratory conditions, is known to show sex differences in survival, where females live longer than males (Tower and Arbeitman, 2009). Intriguingly, forced glycogen synthesis in the neurons reduces survival in *Drosophila* (Duran et al., 2012) and, on the other hand, a neuron-specific GS knockdown extends lifespan (Sinadinos et al., 2014). Therefore, we checked whether changes in brain glycogen levels could have a sex-specific effect on the lifespan of the flies. To address this, we have employed two different approaches: (i) pan-neuronal knockdown of GS using RNAi (*elav>GSRNAi*) and (ii) overexpression of the human wild-type GS (*elav>hMGS-wt*), also used in the previous studies conducted by Duran et al. (2012) and Sinadinos et al. (2014). Transgenic lines overexpressing pan-neuronal GFP (the *elav>GFP*) were used as control (Duran et al., 2012). As shown in Fig. 1A, the *elav>GFP* female flies (75.66±2.06 days) survived longer than the *elav>GFP* males (59.86±4.47 days), in line with the general idea that females outlive males in a population (Tower and Arbeitman, 2009). Contrary to the controls, the *elav>GSRNAi* male flies survived longer (55.34±2.22 days) than the *elav>GSRNAi* females (45.06±2.81 days) (Fig. 1B). Similarly, male flies of the genotype *elav>hMGS-wt* (41.62±1.79 days) survived significantly longer as compared with their female counterparts (22.88±1.31 days), although their lifespan was significantly shorter than the *elav>GSRNAi* males (Fig. 1B). Unlike females, there was no significant difference between the survival of control (*elav>GFP*, 59.86±4.47 days) males and GS knockdown males (*elav>GSRNAi*, 55.24±2.22 days), although the lifespan of GS overexpression males (*elav>hMGS-wt*, 41.62±1.79 days) was shorter as compared to that of the other two genotypes. The mean lifespan, log-rank, and *P* values calculated for the above observations are shown in Table 1. Log-rank (Mantel–Cox) test values of cumulative survival of males and females of *elav>GFP, elav>GSRNAi*, and *elav>hMGS-wt* showed significant differences (Table 1). Significant variations in lifespan between genotypes (*F*=18.762, *P*=0.002) and sexes (*F*=11.793, *P*=0.011) were observed (Table 2). The interaction between genotype and sex was also significantly different (*F*=40.722, *P*=0.0001). The efficiency of GS knockdown and the overexpression of human GS in the two genotypes were confirmed at the transcript and the protein level, respectively (Fig. 1C,D). Thus, we noted that changes in brain glycogen levels significantly alter the lifespan of *Drosophila* females.

**Table 1.**
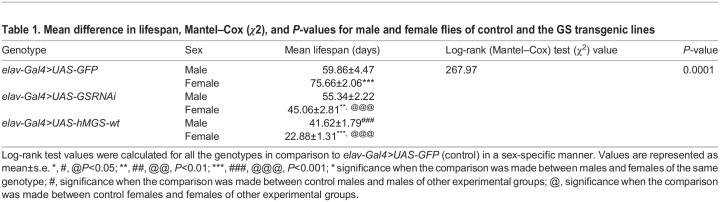
Mean difference in lifespan, Mantel–Cox (*χ*2), and *P*-values for male and female flies of control and the GS transgenic lines

**Table 2.**
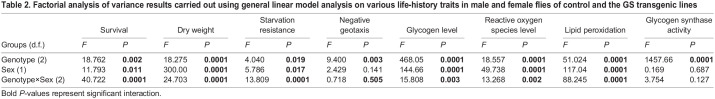
Factorial analysis of variance results carried out using general linear model analysis on various life-history traits in male and female flies of control and the GS transgenic lines

### Effect of brain glycogen alterations on fitness parameters

**Fig. 2.**
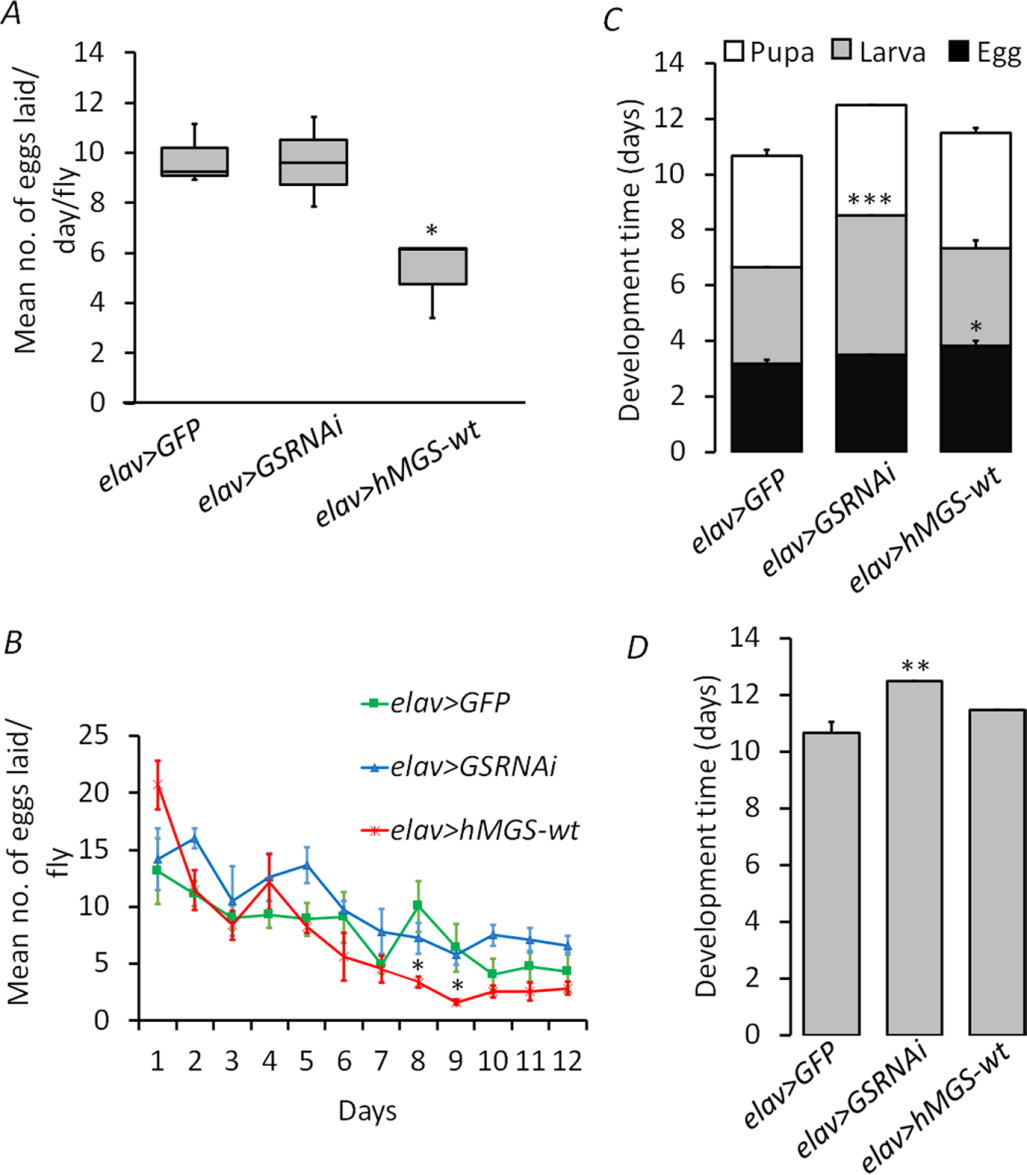
**Effect of brain glycogen alterations on fitness parameters**. Fitness parameters were analyzed in *elav>GFP* (control) and GS transgenic flies (*elav>GSRNAi* and *elav>hMGS-wt*). (A) Average total daily fecundity/fly (*N*=10); (B) Average fecundity per week/fly (*N*=10). (C) Development time (days) for the pre-adult stages (egg, larva, and pupa, *N*=5); (D) Overall development time (days, *N*=5). Each value represents the mean±s.e.; *P<0.05; **P<0.01; ***P<0.001.

Our observations with the GS knockdown and GS overexpression lines are contrary to the general observations that females live longer than males across species. One possible explanation for this could be the disposable soma theory of aging, wherein a trade-off in energy allocation between reproduction and survival is proposed (Kirkwood, 1977). Therefore, we looked at the role of brain glycogen levels on fecundity as a measure of reproduction in flies. Here, we looked at the fecundity for all the lines used in the study i.e., *elav>GFP, elav>GSRNAi*, and *elav>hMGS-wt* (Fig. 2A,B). The GS-knockdown line (*elav>GSRNAi*, 9.63±1.02 eggs/day) had no significant difference as compared to the controls (*elav>GFP*, 9.78±0.51) in the average number of eggs laid per day (Fig. 2A). For the *elav>hMGS-wt* line, however, the fecundity (5.22±0.91 eggs/day) was significantly lower as compared to the control group (*elav>GFP*, 9.78±0.51) (Fig. 2A). The *elav>hMGS-wt* line showed higher progeny numbers during their early part of the adult stage as compared to the other genotypes, which significantly declined in the later life (Fig. 2B).

During the life cycle of *Drosophila*, the larval stage appears to acquire maximum energy resources (Partridge and Fowler, 1992). Both larval development and pre-adult survival are crucial for the overall fitness in *Drosophila*. Therefore, we investigated whether changes in brain glycogen levels could affect development time in the flies. For this, we assessed the overall development time (Fig. 2C) across all the genotypes. The *elav>GSRNAi* flies showed extended development time (12.5±0.00 days) as compared to the *elav>GFP* (10.6±0.44 days) (Fig. 2D), primarily due to the increased time spent in the larva stage (Fig. 2C). However, the *elav>hMGS-wt* line did not show such a marked difference though they seem to spend more time in development as eggs.

To explore the possible link between lifespan and body weight, we measured the dry weight of flies belonging to various genotypes. As shown in Table 2, dry weight varied significantly between the genotypes (*F*=18.275, *P*=0.0001) and sexes (*F*=300.0, *P*=0.0001). Statistical analysis showed significant interactions between genotype×sex for the dry weight (*F*=24.703, *P*=0.0001) (Table 2). Females were heavier than males irrespective of the genotypes (Fig. S1). The dry weight of the control (*elav>GFP*) females was significantly higher (1.8±0×10^−3^g) when compared to the GS knockdown (1.2±0×10^−3^g) and GS overexpression (0.9±0×10^−3^g) females. There was no significant difference in the weight of males across the experimental genotypes [(*elav>GFP*, 0.44±0×10^−3^ g); (*elav>GSRNAi*, 0.36±0×10^−3^ g); (*elav>hMGS-wt*, 0.48±0×10^−3^ g) (Fig. S1)].

### The sex-specific difference in brain glycogen content correlates with female-specific resistance to starvation

**Fig. 3.**
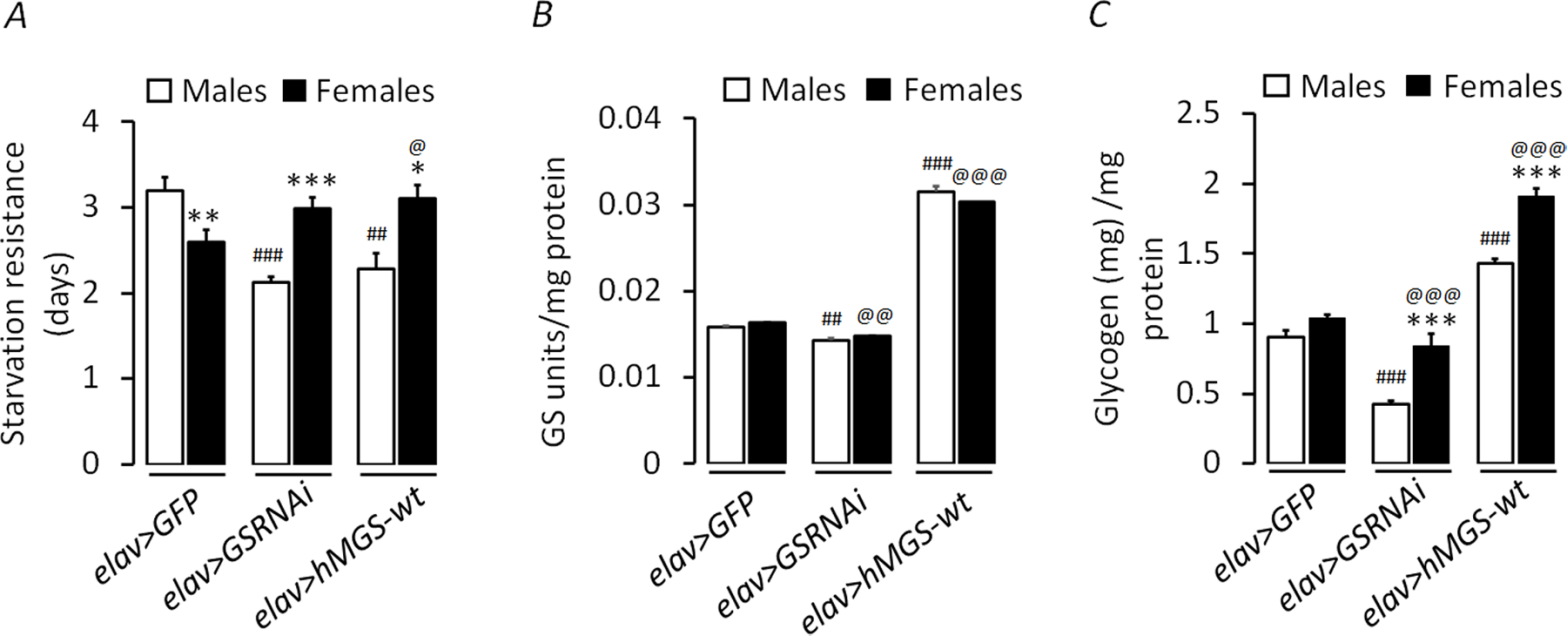
**Modulation of brain glycogen content correlates with female-specific resistance to starvation.** (A) Bar diagram showing absolute starvation resistance in male and female flies of *elav>GFP* (control) and GS transgenic lines (*elav>GSRNAi* and *elav>hMGS-wt, N*=5)*.* Bar diagram showing sex-specific GS enzyme activity (*N*=6) (B) and glycogen levels (*N*=6) (C) of *elav>GFP* (control) and GS transgenic flies (*elav>GSRNAi* and *elav>hMGS-wt*)*.* Each value represents the mean±s.e. ^*^, ^#^, ^@^*P*<0.05; ^**^, ^##^, ^@@^*P*<0.01; ^***^, ^###^, ^@@@^*P*<0.001. *, significance when the comparison was made between males and females of the same genotype; #, significance between control males and males of other experimental groups; @, significance between control females and females of other experimental groups.

The nutritional challenge is known to affect both survival and reproduction in organisms. In continuation of the two fitness traits studied, we further examined the response of prolonged starvation on flies with variable levels of brain glycogen, given that glycogen serves as an energy reserve. For this, we measured resistance to starvation for both knockdown (*elav>GSRNAi*) and overexpressed (*elav>hMGS-wt*) models of GS and in both the sexes (Fig. 3A). There was a significant variation in starvation resistance between genotypes (*F*=4.040, *P*=0.019) and sexes (*F*=5.786, *P*=0.017) as well as the interaction between genotype x sex was found significant (*F*=13.809, *P*=0.0001) (Table 2). In the control flies, starvation resistance was higher in males than females, whereas in GS transgenic flies, females displayed higher starvation resistance than males. Based on our results, starvation resistance appears to negatively correlate with the lifespan, wherein short-lived female flies of *elav>GSRNAi* and *elav>hMGS-wt* were resilient to starvation stress as compared to their long-lived male sex.

Moreover, the inverse relationship in starvation resistance observed between the control and GS transgenic flies indicated that changes in brain glycogen levels have a pronounced sex-specific effect on physiology. We hypothesized that a difference in glycogen content between males and females might underlie this effect. Therefore, we wanted to check whether females store a higher amount of glycogen compared to their male counterparts. For this, we estimated the GS enzyme activity and the glycogen levels in the fly heads of all genotypes at one week of age (Fig. 3B,C). Measurements of both glycogen and GS activity were carried out in a sex-specific manner. One-way ANOVA was used to calculate the significance across genotypes. The *elav>GFP* (control) flies had a negligible and non-significant difference in their GS activity and glycogen values between sexes (Fig. 3B,C). On the other hand, in the other two genotypes (*elav>GSRNAi* and *elav>hMGS-wt*), the females had considerably higher glycogen reserves when compared to males (Fig. 3C), though there was no significant difference in the GS enzyme activity between sexes (Fig. 3B). As expected, the GS activity and the glycogen content in both the sexes were lowest in the GS knockdown flies (*elav>GSRNAi*) and the highest in the flies overexpressing the wild-type form of GS (*elav>hMGS-wt*) as compared to the controls (*elav>GFP*). Statistical analysis revealed the significant interactions for glycogen level between genotypes (*F*=468.05, *P*=0.0001), sexes (*F*=144.06, *P*=0.0001) and genotype x sex (*F*=1.808, *P*=0.0001) (Table 2). Nevertheless, the interactions for GS activity between genotypes was significant (*F*=1457.66, *P*=0.0001), but non-significant between sexes (*F*=0.169, *P*=0.687) and genotype x sex (*F*=3.754, *P*=0.127). Thus, higher starvation resistance in the females of both GS-knockdown and for the GS-overexpressed lines correlates with their altered glycogen reserves. Therefore, female flies with altered brain glycogen (either low or high) show a greater defense against starvation throughout their life when compared to males.

### Effect of brain glycogen on oxidative stress and lifespan in females

**Fig. 4.**
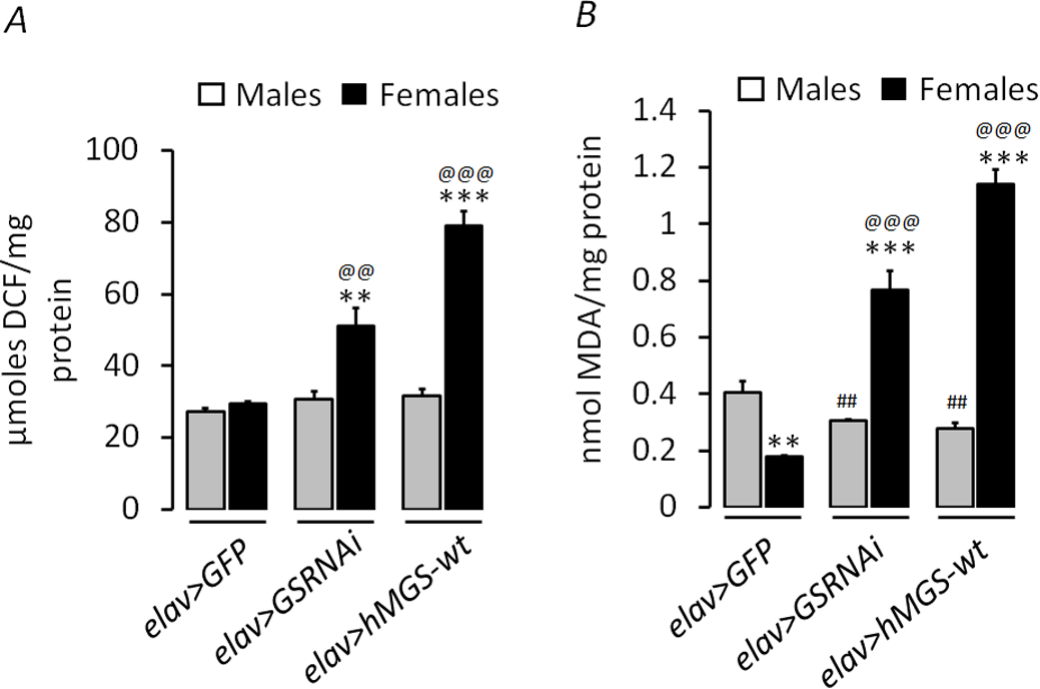
**Elevated brain glycogen levels correlate with increased oxidative stress in female flies.** (A) Bar diagram showing the reactive oxygen species (ROS) levels in the brain of male and female flies of *elav>GFP* (control) and GS transgenic lines (*elav>GSRNAi* and *elav>hMGS-wt, N*=6)*.* (B) Level of malondialdehyde (MDA) equivalents (as a measure of lipid peroxidation) in male and female flies of *elav>GFP* (control) and GS transgenic lines (*elav>GSRNAi* and *elav>hMGS-wt, N*=6)*.* Each value represents the mean±s.e. ^*^, ^#^, ^@^*P*<0.05; ^**^, ^##^, ^@@^*P*<0.01; ^***^, ^###^, ^@@@^*P*<0.001. *, significance when the comparison was made between males and females of the same genotype; #, significance between control males and males of other experimental groups; @ significance between control females and females of other experimental groups.

Glycogen accumulation has been observed in the neurons subjected to various stressors, including oxidative stress (Saez et al., 2014; Wit et al., 2013; Puri et al., 2011; Onkar et al., 2020). Moreover, the lifespan of organisms is inversely correlated with the increased oxidative damage and ROS levels (Sohal and Orr, 2012). Given the female-specific decrease in lifespan and increased glycogen reserves and the established correlation of glycogen build-up in the neurons under stress conditions, we hypothesized that a shorter lifespan and/or decreased survival observed in the females might result from higher oxidative stress in the female flies. Thus, we wanted to measure the possible sex-specific difference in the oxidative stress levels in flies of all genotypes used in the present study. For this, we used the DCFDA method (Black and Brandt, 1974) to check the difference in the relative levels of ROS and thiobarbituric acid reactive substance (TBARS) assay (Ohkawa et al., 1979) to measure lipid peroxidation levels, as oxidative stress markers, between males and females (Fig. 4A,B). Notably, the oxidative stress level indeed differed between males and females of the *elav>GSRNAi* and *elav>hMGS-wt* genotypes. In the DCFDA estimation, females were found to have a significantly higher amount of ROS levels as compared to males in the GS knockdown (*elav>GSRNAi)* and overexpression (*elav>hMGS-wt*) states (Fig. 4A). This difference was absent in the control *elav>GFP* males and females. There was a significant variation in ROS levels between genotypes (*F*=18.557, *P*=0.0001) and sexes (*F*=49.738, *P*=0.0001) as well as the interaction between genotype x sex was found significant (*F*=13.268, *P*=0.002) (Table 2). In line with the ROS levels, lipid peroxidation in the TBARS assay was found to be higher in the *elav>GSRNAi* and *elav>hMGS-wt* females with increased brain glycogen levels compared to males (Fig. 4B). Contrastingly, in control flies, males showed higher levels of malondialdehyde (MDA) compared to females. There was a significant variation in lipid peroxidation between genotypes (*F*=51.024, *P*=0.0001) and sexes (*F*=117.04, *P*=0.0001) as well as the interaction between genotype x sex was found significant (*F*=82.245, *P*=0.0001) (Table 2). We attribute the marked increase in oxidative stress and altered brain glycogen variations to the shortening of lifespan in females compared to the male flies.

### Effect of brain glycogen on locomotor activity

Negative geotaxis is used as an index of locomotor behavior in flies (Feany and Bender, 2000). The loss or impairment in climbing ability is used as a readout of motor changes. Thus, to determine whether or not the brain glycogen level will affect motor behavior, we monitored the locomotory performance of flies representing various genotypes used in the study (Fig. S2). We observed reduced locomotor activity in GS-overexpressed flies (*elav>hMGS-wt*) when compared to other genotypes. Statistical analysis showed a significant variation between genotypes (*F*=9.400, *P*=0.003), whereas interactions between sexes (*F*=2.429, *P*=0.141) and genotype x sex (*F*=0.718, *P*=0.505) were non-significant (Table 2).

## DISCUSSION

Although a correlation between glycogen levels and lifespan has been established in fly and mouse models where a lower GS activity enhances lifespan *(elav>GSRNAi*), and a higher enzyme activity resulted in a shorter lifespan (*elav>hMGS-wt*) (Duran et al., 2012; Sinadinos et al., 2014), the sex-specific effect of glycogen alterations in survival and other fitness traits has not been investigated yet. This study demonstrated that female flies with altered brain glycogen levels exhibit a shortened lifespan and higher sensitivity to oxidative stress than males. Further, altered glycogen levels had a positive effect on starvation resistance in female flies as compared to their male counterparts. Our findings offer a novel concept on brain glycogen-mediated control of sex-specific traits, which is beyond the well-known function of glycogen being the energy pool of the cell.

Notwithstanding the concept of the females being the longer-lived sex in *Drosophila* (Tower and Arbeitman, 2009; Niveditha et al., 2017), we observed males living longer than females in transgenic flies having an altered brain-specific GS activity. Differential regulation of GS, or GS-regulated pathways, in the two sexes could possibly be one of the possible reasons that underlie this response where the activity of GS might be optimized in females in comparison to males. Thus, an imbalance in the GS activity and/or glycogen levels abolished the female advantage in survival over males in our study. Such differential expression of the transcriptome is indeed reported in various models wherein the expression of certain genes is antagonistically selected in one sex, and their altered expression could be deleterious for the other (Ellegren and Parsch, 2007; Rice, 1996; Ranz et al., 2003). However, it is intriguing to note here that the partial knockdown and the overexpression of GS showed a similar trend for some of the phenotypes analyzed. Indeed, either the loss or gain of some of the neuronal proteins involved in homeostatic processes are known to result in similar neuronal defects (Winklhofer et al., 2008; Kabashi et al., 2010; Chung et al., 2020). We did not find a difference in the lifespan between the males of control (*elav>GFP*) and GS knockdown (*elav>GSRNAi*), however, the male lifespan decreased with GS overexpression (*elav>hMGS-wt*). As the pattern for male lifespan was not converse or parallel in the two genetic perturbations as compared to the control, we reasoned that altered brain glycogen does not influence male lifespan and the specific decrease of male lifespan in the GS overexpression line is because of the fact that glycogen accumulation leads to premature aging in flies as shown previously (Duran et al., 2012; Sinadinos et al., 2014).

In our study, the fecundity of the flies remained unaltered in the GS knockdown line (*elav>GSRNAi*) but reduced in GS overexpressed line (*elav>hMGS-wt*), suggesting a lack of significant role in survival. However, we correlate the uncompromised fecundity with a shorter lifespan of females as demonstrated by Zwaan et al. (Zwaan et al., 1995). Reduction in the fecundity of *elav>hMGS-wt* flies could be attributed to early reproductive benefits, wherein these flies showed a higher fecundity rate during the initial days of lifespan that eventually plateaued. In addition, we observed an extension in the overall development time in the GS transgenic flies indicating a prolonged feeding time in these genotypes, which might help in enhanced adult fitness as shown earlier (Prasad and Joshi, 2003). The longer feeding time during development might help in allocating resources for increased weight, especially in female flies. Increased body weight has been correlated with a longer lifespan (Partridge and Fowler, 1992). Although control flies showed a positive correlation between body weight and longer lifespan in our study, we found a negative relationship in experimental groups.

The ability to survive food shortage is an adult fitness trait and starvation resistance is specifically reported in *Drosophila* as an adaptive trait orchestrated by many genes (Goenaga et al., 2013). Emerging reports describe that women live longer than men during severe famine (Zarulli et al., 2018). In agreement with previous studies, female flies of the GS transgenic lines displayed an increased starvation resistance as compared to the males and this might correlate to the increase in the glycogen content in GS transgenic females. However, the *elav>hMGS-wt* males displaying decreased starvation resistance and the higher glycogen content suggests that glycogen might not be the primary reservoir of energy in male flies during starvation. A difference in glycogen use upon starvation between genders is indeed reported in the literature (Aggarwal, 2014; Chauhan et al., 2021). Recently, Yamada et al. (2019) have also shown higher starvation resistance in female GS-null mutants compared to male flies. Glycogen here can act as an energy pool for females under starvation similar to our observation. A decrease in the brain glycogen content upon starvation and a compromised starvation resistance were reported in these GS mutants with fluctuating dietary carbon (Yamada et al., 2019), further confirming that glycogen reserves in the brain facilitate starvation stress. Furthermore, knocking down GS is shown to extend lifespan in *C. elegans*, flies, and mice (Magwere et al., 2004; Duran et al., 2012; Gusarov et al., 2017). In line with the literature, our results also reveal that lower GS activity enhances lifespan (*elav>GSRNAi*), whereas higher enzyme activity results in a shorter lifespan (*elav>hMGS-wt*). Brain glycogen metabolism is also suggested to be sexually dimorphic (Tamrakar et al., 2014), as gonadal steroids are known to affect brain and body bioenergetics (viz., glucose uptake, mitochondrial oxidative phosphorylation, oxidative stress, etc.) (Rettberg et al., 2014). This could underlie the enhanced storage of glycogen in females as compared to males observed in the present study. Another fitness index of the present study, the negative geotactic behavior, which mostly depends on age, sex, and oxidative stress (Deepashree et al., 2019), did not show a prominent sex-specific change in our study, suggesting a lack of glycogen-mediated influence on gender-specific locomotion.

Oxidative damage to the biomolecules contributes to the sex-specific shortening of lifespan, which could be due to the malfunctioning of the mitochondria (Tower and Arbeitman, 2009) or increased metabolism (Archer et al., 2013). Oxidative stress remains one of the key factors in aging and age-related neurodegenerative disorders like Alzheimer's and Parkinson's diseases. Furthermore, lipid peroxidation is regarded as the major process causing oxidative damage, and age-associated sex-specific elevation in lipid peroxidation is also reported (Rikans and Hornbrook, 1997). Under normal physiological conditions, females are less susceptible to oxidative stress due to lower levels of NADPH-oxidases, higher antioxidant potential, and female-specific hormones like estrogen (reviewed in Kander et al., 2017). In contrast to previous studies, GS transgenic female flies showed higher ROS and MDA levels compared to males. In agreement with earlier studies, the higher ROS/MDA level is associated with decreased survival. Additional work demonstrating antioxidant defenses (superoxide dismutase and catalase) in both sexes may provide a clear picture to substantiate the difference in ROS homeostasis and its role in survival. Nonetheless, our results indicate that brain glycogen alterations might influence the ROS levels possibly by affecting mitochondrial function. Similarly, the glycogen content in the brain may also modify the neuronal circuitry involved in sex-specific hormonal regulation. For example, estrogen lengthens the lifespan in females because it possesses antioxidant and anti-inflammatory properties (Barja, 2004; Mann et al., 2007). This property of estrogen also extends to the brain, as it has a neuroprotective effect, and loss of estrogen increases ROS levels and accelerates aging in diseases like Alzheimer's disease (Stice et al., 2009). Malfunctioning of the hormonal axis due to a change in brain glycogen ratios can impact the hormonal profile, increasing the pro-oxidant levels, particularly in the female flies. A concurrent role between glycogen and pro-oxidants for regulating lifespan differences is also evident in our study, wherein flies with low pro-oxidants and glycogen survived longer than flies with higher pro-oxidants and glycogen levels. Taken together, we propose that these fitness traits act synergistically to help the organism survive better against the odds and a direct correlation exists between brain glycogen alterations, specifically in the females, and pathways that regulate adult fitness traits. Thus, glycogen homeostasis in the fly brain appears to be essential for maintaining female physiology and normal health span.

## MATERIALS AND METHODS

### Materials

Amyloglucosidase, 2′,7′-dichlorodihydrofluorescein-diacetate (DCF-DA), bovine serum albumin was procured from Sigma-Aldrich Chemicals India Pvt Ltd. All the other chemicals used in the present investigations were of analytical grade.

### Transgenic fly stocks and culture

*UAS-GFP* and the pan-neuronal driver line *elav-Gal4^C155^* was a kind gift from Prof. Pradip Sinha (IIT Kanpur, India). Transgenic fly for the wild-type form of hMGS (human Muscle Glycogen Synthase), *UAS-hMGS-wt* (wild-type form of the hMGS), and *UAS-GSRNAi* (v35136) was a kind gift from Professor Joan J. Guinovart (IRB, Barcelona, Spain) and is reported in a previous study (Duran et al., 2012; Sinadinos et al., 2014). Males of the *UAS-hMGS-wt* and *UAS-GSRNAi* were crossed with the virgins of *elav-Gal4^C155^* to drive their expression pan-neuronally (Yao et al., 1993). *UAS-GFP* transgene driven with *elav-Gal4^C155^* served as a control.

Flies were maintained at a density of ∼100 flies in 40 ml cornmeal–agar medium per 200 ml volume glass bottle at 22±1°C under a constant light-dark cycle with 70%-80% humidity. Following eclosion, flies were aged for 3-4 days and transferred to fresh bottles with media containing live yeast for serial cultures. During experiments, ∼20 flies in 5 ml cornmeal–agar medium per 40 ml volume glass vials were employed.

### Lifespan analysis

Freshly eclosed flies were collected, sexed, and transferred (on the same day of eclosion) to glass vials containing 10 ml cornmeal–agar medium. The flies were transferred to fresh food vials every alternate day for 60 days and thereafter for every 3-5 days depending on media condition. Flies dying during the course of lifespan assessment were not replaced. The survival of flies was monitored daily until all the flies died (Sharmila Bharathi et al., 2003). For each genotype and sex, five vials were set up with 20 flies in each. The mean lifespan of flies was calculated for all the groups.

### Fecundity

Freshly eclosed virgin flies were collected, sexed, and transferred (on the same day of eclosion) to separate glass vials containing 10 ml cornmeal–agar medium. To determine fecundity, an unmated male and a virgin female were introduced in the media vial. This pair was transferred to a fresh media vial every day and the eggs laid by females in the preceding day were counted using a stereomicroscope. Fecundity assay was carried out for 12 days; while the dead males were replaced in the course of this assay. A separate group of male flies obtained from the same batch of cultures was maintained in parallel for this purpose (Sharmila Bharathi et al., 2003). Ten vials were set up for each line. The total number of eggs laid by a female in a day for a given period of time (average daily fecundity) and the fecundity per week/fly were calculated from the data thus collected.

### Development time

For assessing the durations of egg-to-adult developmental stages (egg, larva, and pupa), 20 eggs of the same age were placed in a culture vial. These vials were maintained in the vivarium at 22±1°C under a constant 12:12 light-dark cycle with 70%-80% humidity. The time to complete each developmental stage was recorded via regular observation of vials until there was no eclosion of flies for two consecutive days. Five such vials were set up for each genotype and sex. The time taken (days) by eggs to hatch, the larvae to pupate, or the pupae to eclose was recorded for all the genotypes (Wit et al., 2013).

### Dry weight

Five males or females in 10 batches for each genotype were included in the measurement of dry weight. The flies were transferred to an Eppendorf tube, euthanized by freezing, dried for 36 h at 60°C, and weighed (Sharmila Bharathi et al., 2003).

### Starvation resistance

Freshly eclosed flies were collected; the males and females were separated and allowed to age for seven days. Ten males and ten females were transferred to separate vials containing non-nutritive media (7 ml of 1% agar only). The vials were plugged with cotton and maintained in a vivarium at 22±1°C under 12:12 light-dark cycles. The flies in these vials were monitored for mortality every 2-3 h until all flies died. The number of flies alive against starvation (in days) between genotypes was monitored until all flies died. Five replicates for each sex and genotype were set up for the assay. The absolute starvation resistance of each group (genotypes and sexes) was calculated as the time until death (in h) for each fly (Sharmila Bharathi et al., 2003).

### Glycogen estimation

The glycogen content in the fly head was measured using the protocol described earlier (Rai et al., 2018) with modifications. Briefly, 20 fly heads per replicate for each genotype and sex were homogenized in 120 µl of 30% KOH solution and boiled at 100°C for 20 min. Twenty microliters of this homogenate were used for protein quantification, while the remaining 100 µl was spotted on a 2 cm×2 cm Whatman filter paper (#31-ET CHR). The spotted sample was given three consecutive 66% ethanol washes and dried overnight. The dried filter paper was further treated with the enzyme amyloglucosidase (0.5 mg/ml in 0.2 M sodium acetate buffer, pH 4.8) to release glucose. This free glucose was measured using the glucose colorimetric assay kit (ERBA Diagnostics Mannheim Gmbh Ltd) and was normalized to the protein values and plotted as fold change. Six replicates were taken for each sex and genotype for the assay.

### Glycogen synthase activity

Glycogen synthase activity was estimated by modifying Danforth's spectrophotometric method (Danforth, 1965). Briefly, 50 fly heads per replicate for each genotype and sex were homogenized in 100 µl of phosphate-buffered saline. The homogenate was added to a reaction mixture containing 48 mM Tris (pH 8.2), 12.4 mM MgCl2, 1 mM EDTA, 2.4 mM 2-mercaptoethanol, 3.63 mM UDPG, 9.7 mM glucose 6-phosphate and was incubated for 5 min at 30°C. The reaction was terminated by boiling the mixture for 5 min. An increase in absorbance at 340 nm with the addition of enzymes was measured for 5 min and was normalized to the total protein content.

### Reactive oxygen species measurement

Reactive oxygen species were measured using the DCF-DA method (Black and Brandt, 1974). The reaction mixture consisted of Tris-HCl buffer (0.1 M, pH 7.4), 100 µl fly head homogenate, and 10 µM DCF-DA. The reaction mixture was incubated for 30 min at room temperature. The breakdown of DCF-DA to the fluorescent product 2′,7′-dichlorofluorescein (DCF) was measured in a microplate reader (Spectrofluorometer) with an excitation wavelength of 488 nm and emission at 525 nm. The values were plotted against the standard DCF graph and expressed as µmol DCF formed/min/mg protein.

### Lipid peroxidation

Lipid peroxidation was measured by TBARS assay (Ohkawa et al., 1979). Briefly, the reaction mixture contained 1.5 ml acetic acid (20%), 250 µl fly homogenate, 1.5 ml of TBA (0.8% w/v), and 200 µl sodium lauryl sulphate. The mixture was incubated in a boiling water bath for 45 mins and extracted into 3 ml of 1-butanol. The absorbance was measured at 535 nm and quantified as malondialdehyde equivalents.

### Negative geotactic behavior

The inherent behavioral response to climb (negative geotaxis) of flies has been employed to assess their locomotor ability. Ten flies for each genotype and sex with five replicates were employed. Flies were transferred to a vertical column of 25 cm in length and a diameter of 1.5 cm. The flies were gently tapped thrice to the bottom of the column, and the flies that reached the top of the column and those that remained at the bottom were counted separately. The results were expressed as the number of flies that escaped beyond the minimum distance in the 20 s interval (Feany and Bender, 2000).

### RNA extraction and real-time PCR

Total RNA was isolated from 20-25 fly heads of each genotype using the Trizol reagent according to the manufacturer's protocol (Life Technologies India). cDNA was synthesized from 1 µg of total RNA using random primers and the efficacy of reverse transcription was verified by amplification of the housekeeping gene *Rpl32* and the transcript level for glycogen synthase was measured using *Drosophila* specific primers (*GlyS*). Real-time PCR was performed using the Luna Universal qPCR master mix (New England BioLabs) and a CFX-96 real-time PCR machine (Bio-Rad Laboratories, India). The primer sequences used for the PCR amplification are *Rpl32*-F ATCGGTTACGGATCGAACAA and *Rpl32*-R GACAATCTCCTTGCGCTTCT; *GlyS (Drosophila)*-F GTTATTCGTTTTGTTTCGTGTGGC and *GlyS(Drosophila)*-R CGAGCGCAATGAGTTGACAG.

### Immunoblotting

Western blot analysis for checking the expression of GS in the fly heads was performed as previously described (Rai et al., 2018). Briefly, 20 fly heads for each genotype were pooled and lysed in Laemmli buffer and the protein extracted was estimated using the BCA method. Equal amounts of protein were loaded in a 10% SDS-PAGE and transferred to the nitrocellulose membrane (Biorad, 1620112). The membranes were then blocked with 5% nonfat dry milk powder (Biorad, 1706404) in 1X TBST and probed for the anti-GS antibody (Cell Signalling Technology, #3893, 1:1000) overnight (Rai et al., 2018). The blot was then probed for the recommended secondary antibodies and the immunoreactive bands were detected with a chemiluminescent detection kit (Supersignal West Pico, Pierce). The signal intensity of γ-tubulin (Sigma-Aldrich Chemicals India Pvt Ltd., T6557, 1:10,000) served as the loading control.

### Protein estimation

Protein estimation was carried out using the BCA method (Smith et al., 1985) with bovine serum albumin as a standard.

### Statistical analyses

Statistical analyses of the data were carried out using SPSS software (Version 17.0, SPSS Inc., Chicago, IL, USA). For survival assay, Kaplan–Meier survivorship curves and log-rank (Mantel–Cox) test values were used for calculating significance. Data for all parameters (survival, dry weight, starvation resistance, negative geotaxis assay, and biochemical investigations) were subjected to general linear model analysis using factorial analysis of variance (ANOVA) with sex and genotype groups as fixed factors and individual values as dependent factors. The multiple comparisons were carried out by LSD's post-hoc test. Statistical significance for real-time analysis was subjected to a two-tailed unpaired *t*-test using the Graphpad Prism software (Version 7, GraphPad Software Inc., California). *P*-value <0.05 is considered as significant.

## Supplementary Material

Supplementary information
