# Supplementary material for: Alterations in brain glycogen levels influence life-history traits and reduce the lifespan in female Drosophila melanogaster
Source: Biol Open. 2021 Dec 14;10(12):bio059055. doi: 10.1242/bio.059055 (PMC8689487; doi:10.1242/bio.059055)
Supplement: Supplementary information [file biolopen-10-059055-s1.pdf]

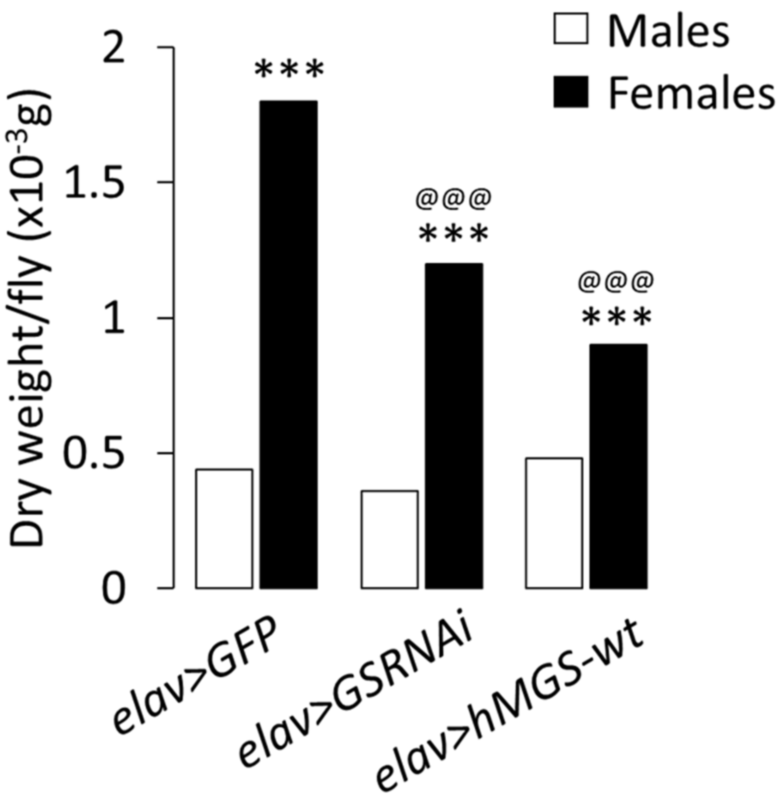


**Fig. S1.** Dry weight (in x$10^{-3}$ g per fly) in male and female flies of *elav>GFP* (control) and GS transgenic lines (*elav>GSRNAi* and *elav>hMGS-wt*), N=10. Each value represents the mean± S E . $^{*,\ @}$ P<0.05; $^{**,\ @@}$ P<0.01; $^{***,\ @@@}$ P<0.001. * denotes the significance when the comparison was made between male and female of the same genotype; @ denotes the significance between control females and females of other experimental groups.

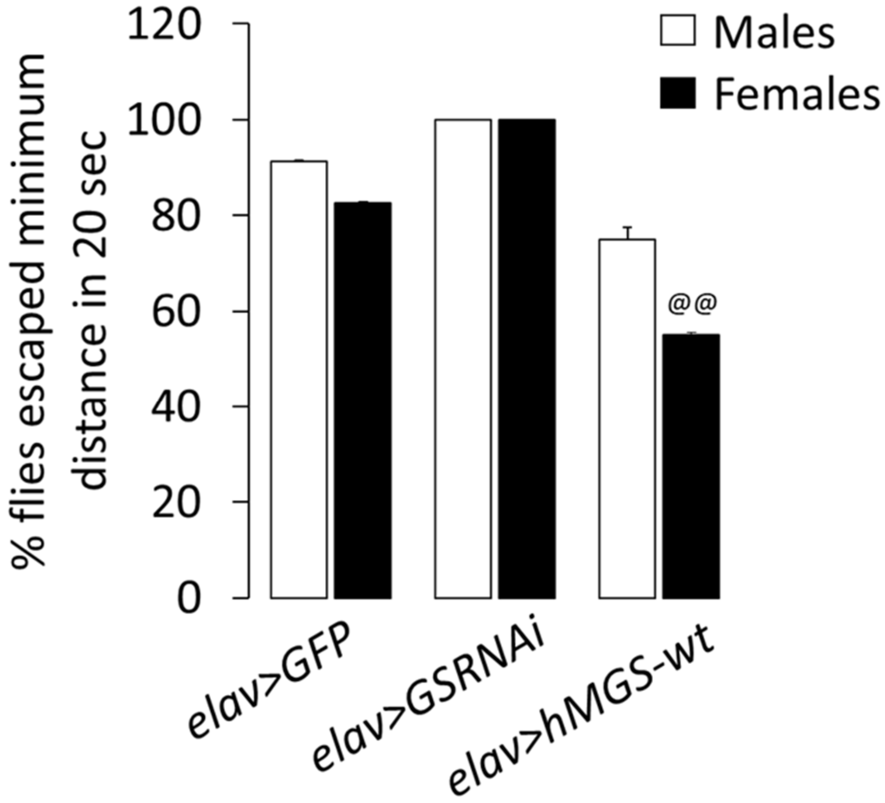


**Fig. S2.** Locomotor ability (negative geotactic behavior) in male and female flies of *elav>GFP* (control) and GS transgenic lines (*elav>GSRNAi* and *elav>hMGS-wt*), N=5. Each value represents the mean ± SE. @@ P<0.01; @ denotes the significance between control females and females of other experimental groups.
